# Case Report: A giant uterine leiomyoma with pseudo-Meigs syndrome combined with a triad of umbilical hernia-prolapse-thrombosis

**DOI:** 10.3389/frph.2026.1790732

**Published:** 2026-04-10

**Authors:** Wenji Luo, Jingjing Shen, Zhihua Liu, Changzhong Li, Yunfei Wang

**Affiliations:** 1 Center of Obstetrics and Gynecology, Peking University Shenzhen Hospital, Shenzhen, China; 2 Institute of Obstetrics and Gynecology, Shenzhen PKU-HKUST Medical Center, Shenzhen, China; 3 Shenzhen Key Laboratory on Technology for Early Diagnosis of Major Gynecologic Diseases, Shenzhen, China

**Keywords:** giant uterine leiomyoma, pelvic floor dysfunction, pseudo-Meigs syndrome, pulmonary embolism, umbilical hernia

## Abstract

This report describes a rare and complex case in which a patient, due to personal beliefs, persistently refused surgery, allowing a uterine leiomyoma to grow to 27.5 × 19.1 × 23.1 cm. This led to a series of life-threatening complications, including pseudo-Meigs syndrome (PMS) with massive pleural and ascitic effusions, 90% atelectasis of the right lung, left lower limb deep vein thrombosis (DVT) and pulmonary embolism (PE), an umbilical hernia, and severe pelvic organ prolapse (POP). Through multidisciplinary team (MDT) collaboration and a staged management strategy—involving initial inferior vena cava filter placement, nutritional support, and drainage of pleural and ascitic fluid, followed by successful tumor resection—the patient recovered. Postoperative pathology confirmed a cellular leiomyoma. This case profoundly illustrates the “cascade effect” whereby a massive pelvic mass, through mechanical compression and a sharp increase in intra-abdominal pressure, can lead to multi-system dysfunction.

## Introduction

Uterine leiomyomas, the most common benign tumors of the female reproductive system, have a significant epidemiological profile, with a prevalence reaching 20%–50% in women of reproductive age (1). The vast majority are small, slow-growing, or asymptomatic and typically require only regular observation and follow-up. However, when a leiomyoma remains untreated and grows to a giant size (typically defined as diameter >10 cm), it may transcend being a mere local space-occupying lesion. Through direct mechanical compression and secondary systemic pathophysiological changes, it can trigger a series of rare and life-threatening complications, making therapeutic strategy highly challenging (2). For symptomatic uterine leiomyomas, particularly those causing anemia (e.g., chronic blood loss due to menorrhagia) or pressure symptoms (e.g., urinary frequency, constipation, pelvic pain, or abdominal distension), surgical intervention—such as myomectomy or hysterectomy—is the primary treatment modality. Pharmacological therapy (e.g., gonadotropin-releasing hormone agonists) is typically employed as a preoperative adjunct to temporarily reduce fibroid volume or correct anemia, rather than as a curative measure, and does not play a curative role in the management of uterine leiomyomas.

Pseudo-Meigs syndrome (PMS) is one such rare complication, characterized by “benign pelvic tumor (typically ovarian fibroma/thecoma, etc.) accompanied by pleural effusion and ascites, which resolve rapidly after tumor resection”, with an incidence of less than 0.5% (3). The core distinction from classic Meigs syndrome lies in the non-specific nature of the primary tumor, and reports of uterine leiomyoma as the etiology of PMS are relatively uncommon. Even rarer is that the sustained and sharp increase in intra-abdominal pressure caused by a giant pelvic mass may further trigger a complex “cascade effect”. This effect is not limited to the formation of pleural effusion but may, through multiple biomechanical mechanisms, synergistically lead to the disruption of pelvic support structures (e.g., severe pelvic organ prolapse), defects in weakened areas of the abdominal wall (e.g., umbilical hernia), and venous hemodynamic disturbances (e.g., lower extremity deep vein thrombosis and pulmonary embolism). Currently, case reports in domestic and international literature of a single giant uterine leiomyoma simultaneously inducing PMS, a giant umbilical hernia, severe POP, and venous thromboembolism (VTE)—an extreme complex “triad” or “multi-syndrome”—are extremely limited.

This case report aims to present a case of a giant cellular uterine leiomyoma, which grew to approximately 27.5 cm in diameter due to the patient's long-term refusal of treatment. This leiomyoma unusually led concurrently to PMS with massive hydrothorax and ascites causing severe lung atelectasis, left lower extremity DVT and PE, a giant umbilical hernia, and pelvic organ prolapse (POP-Q stage IV), plunging the patient into a critical state of multi-organ dysfunction including respiratory failure, anuria, and cachexia. Through a detailed description of this case, we aim to: Firstly, provide an in-depth analysis of the pathophysiological “cascade reaction” whereby a giant benign pelvic tumor induces multisystem dysfunction through purely physical mechanisms. Secondly, discuss the decision-making process and implementation value of a staged treatment strategy (including preoperative rescue interventions and scheduled tumor resection) under the guidance of a multidisciplinary team (MDT) in such complex and critical cases. Thirdly, emphasize the importance of early identification and active intervention for giant pelvic space-occupying lesions to prevent such catastrophic complications. This report provides valuable experience and reference for the clinical management of similarly extreme and complex cases.

## Case report

A 53-year-old multiparous woman, 2 years postmenopausal, was emergently admitted on February 24, 2025 with acute respiratory failure and anuria. Medical history revealed an 8 cm uterine fibroid identified during routine gynecological ultrasonography four years prior. Despite repeated surgical recommendations, the patient declined intervention due to personal beliefs. During this period of conventional treatment refusal, she abstained from menopausal hormone therapy (MHT) and instead chronically consumed a self-formulated decoction containing Codonopsis pilosula, Angelica sinensis, Ziziphus jujuba, and Dimocarpus longan as herbal supplementation. While these constituents lack hormonal activity, their potential uterine effects require individualized evaluation.

The fibroid demonstrated progressive growth with subsequent ultrasonographic reports describing “ill-defined borders precluding accurate measurement”. Over the past year, she developed progressive abdominal distension and umbilical protrusion without postmenopausal bleeding or acute abdominal pain. Six months prior to admission, a reducible egg-sized mass protruded through the vaginal introitus accompanied by stress urinary incontinence, confining her to prolonged bedrest. Clinical deterioration ensued: dysuria emerged 2 months pre-admission, progressing to dyspnea and dysarthria at 1 month, culminating in anuria and severe bilateral lower limb pain 4 days before presentation. Upon arrival, the cachectic patient was nonverbal.

Medical history was notable for absence of chronic conditions (hypertension, diabetes mellitus), abdominal surgeries, or documented genetic predispositions. During her four-year treatment refusal period, the patient lived alone due to prolonged marital separation. Initial disease exacerbation prompted a distress call to her sister for dyspnea management, though she persistently refused medical evaluation due to resistance to conventional interventions. Ultimately, emergency transport was compelled by family insistence. The patient exhibited marked treatment distrust and resistance during initial hospitalization.

**Figure 1:**
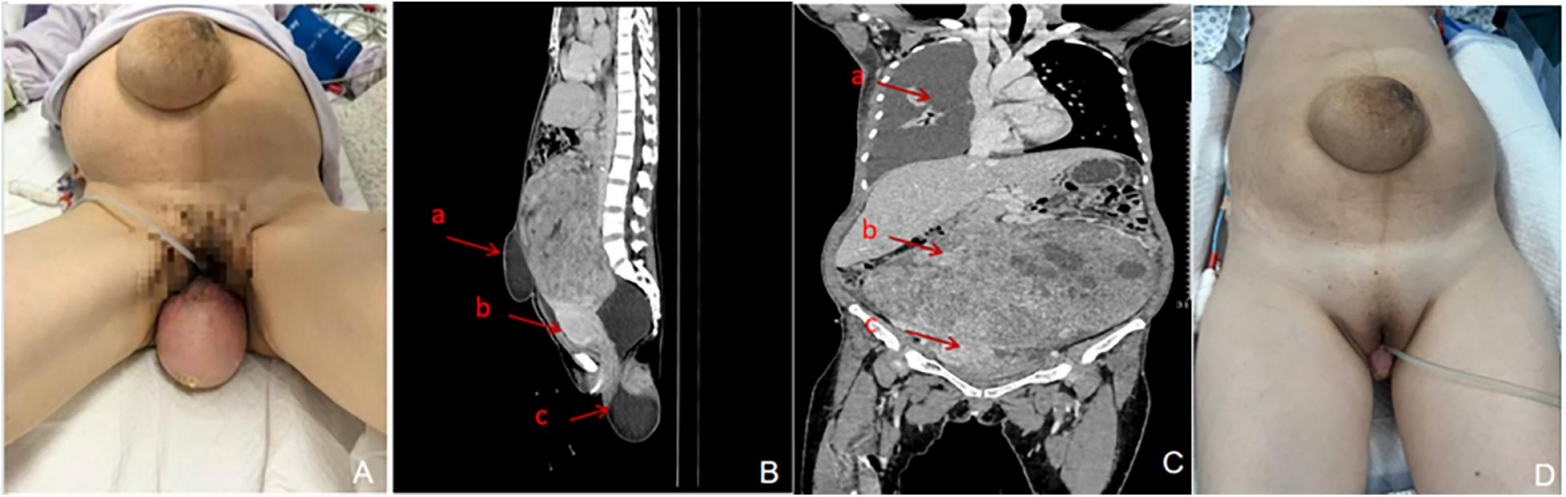
Presentation and imaging findings at admission, and changes after preoperative decompression. **(A)** Supine clinical photograph of the patient on the day of admission, showing marked abdominal distension, a giant umbilical hernia, and posterior vaginal wall prolapse. **(B)** Sagittal reconstruction of an abdominal CT scan obtained at admission. Arrow a indicates the giant umbilical hernia (measuring approximately 121 × 62 × 117 mm); arrow b points to the uterus; arrow c indicates the posterior vaginal wall prolapse (measuring approximately 95 × 90 × 98 mm). **(C)** Coronal reconstruction of a chest CT scan obtained at admission. Arrow a indicates right-sided massive pleural effusion with compressive atelectasis; arrow b points to the huge pelvic mass (later pathologically confirmed as a uterine leiomyoma, measuring approximately 275 × 191 × 231 mm); arrow c marks the normally positioned uterine body. **(D)** Clinical photograph of the patient on the day of surgery (hospital day 25), after completion of the preoperative preparation phase. Compared with panel A, marked improvement in abdominal distension, umbilical hernia, and vaginal prolapse is evident following aggressive drainage and supportive therapy.

Physical and Auxiliary Examination: The patient was emaciated with a markedly distended abdomen resembling a full-term pregnancy. A 12 × 7 × 11 cm umbilical hernia was visible (Figure 1A). Gynecological examination revealed a large protruding mass measuring 10 × 9.5 cm at the vaginal introitus, originating from the posterior vaginal wall. The uterus, cervix, and anterior vaginal wall were normally positioned, while the posterior vaginal wall demonstrated severe prolapse with complete mucosal eversion (Figure 1A). The Pelvic Organ Prolapse Quantification (POP-Q) staging is detailed in Table 1. Vital signs showed tachycardia (125 bpm) and tachypnea (22 breaths/min).

**Table 1:** SCIPOP-Q staging table.

Stage	Aa	Ba	C	Ap	Bp	D	GH (cm)	PB (cm)	TVL (cm)	POP-Q stage
Pre-admission	−3	−3	−8	−3	+10*	−8	5	2	10	IV
Pre-operation	−3	−3	−8	−3	+3	−8	4	2.5	10	III
Post-op immediate	−3	−3	−8	−3	−1	NA	3	3	9	I
1-month follow-up	−3	−3	−8	−3	−2	NA	3	3	9	0–I
3-month follow-up	−3	−3	−8	−3	−2	NA	3	3	9	0
Final follow-up	−3	−3	−8	−3	−2	NA	3	3	9	0

1. Pre-admission Bp ± 10 cm: Estimated based on the spherical prolapse (10 × 9.5 cm). The POP-Q measurement standard uses the hymen as the 0 point, adjusted according to TVL.

2. Post-hysterectomy C point: Represents the vaginal apex position (original cervical C point replaced by vaginal cuff).

3. NA: D point is not applicable after total hysterectomy.

4. GH, genital hiatus; PB, perineal body; TVL, total vaginal length (shortened postoperatively).

Key findings:

1. Plain abdominal CT: A huge mass (approximately 275 mm × 191 mm × 231 mm) was observed in the abdominopelvic cavity, suggestive of a malignant tumor originating from the uterus or adnexa. A subcutaneous cystic hypodense lesion in the lower abdominal wall (approximately 121 mm × 62 mm × 117 mm) communicated with the mass, suggesting possible tumor rupture into the subcutaneous tissue with encapsulation (Figures 1B,C).
2. Chest CT: Right-sided massive pleural effusion with approximately 90% compressive atelectasis of the right lung.
3. Chest ultrasound: Bilateral pleural effusion, measuring approximately 186 mm in depth on the right and 54 mm on the left. The right pleural effusion was superficial (approximately 7 mm from the skin surface) and showed minimal variation with respiration or positional changes.
4. Abdominal ultrasound: A huge solid-cystic mixed echogenic mass in the retroperitoneal space (anteroposterior diameter approximately 200 mm) with ill-defined borders and heterogeneous echotexture. Free intraperitoneal fluid was noted (24 mm anterior to the liver, 72 mm in the right upper quadrant, and 47 mm in the pelvic cavity).
5. Drainage and cytology: Cytological examination of pleural and ascitic fluid obtained via drainage revealed no malignant cells.
6. Vascular imaging: Computed tomography angiography (CTA) and vascular ultrasound confirmed left lower extremity deep vein thrombosis and pulmonary embolism.

Laboratory Tests: Tumor marker CA125 was significantly elevated at 370.0 U/mL, albumin was low at 30.7 g/L, D-dimer was high at 2.55 mg/L FEU, and several coagulation parameters were abnormal (Table 2).

**Table 2:** Dynamic laboratory changes.

Indicator	Baseline (Admission)	Preoperative (Day 25)	Postoperative (Day 5)	Normal range
CA-125 (U/mL)	370.0	285.0	28.5	<35
Albumin (g/L)	30.7	33.7	35.2	35–50
D-dimer (mg/L FEU)	2.55	1.85	0.62	<0.5
PT (s)	15.2	14.5	12.8	11–14
APTT (s)	42	38	30	25–35
Fibrinogen (g/L)	5.8	4.2	3.1	2–4

Faced with this multi-system critical condition, the hospital promptly convened a multidisciplinary team (MDT) involving 13 specialties. Management was divided into two key phases:

The core diagnostic challenge in this case was to attribute a constellation of manifestations—including acute respiratory failure, anuria, a massive pelvic mass, pleural effusion and ascites, thromboembolic events, and pelvic organ prolapse—to a single, treatable etiology. The multidisciplinary team (MDT) adhered to the following diagnostic reasoning: (1). Differentiation of Pseudo-Meigs Syndrome (PMS): The patient presented with the classic combination of a pelvic tumor (giant leiomyoma) accompanied by pleural effusion and ascites, along with markedly elevated CA-125 levels. This necessitated primary differentiation from ovarian malignancy, particularly advanced epithelial ovarian carcinoma with peritoneal and pleural metastases. Key findings supporting PMS/benign etiology included: transudative ascites [high serum-ascites albumin gradient (SAAG)], an extremely high CA-125/CEA ratio, and the absence of systemic evidence of malignancy (e.g., lymphadenopathy, liver metastases). Additionally, other common causes of pleural effusion and ascites—such as cirrhosis, heart failure, and tuberculous peritonitis—were systematically excluded. (2). Differential Diagnosis of the Pelvic Mass: Beyond uterine leiomyoma, considerations included uterine sarcoma, ovarian sex cord-stromal tumors (e.g., fibroma), or gastrointestinal stromal tumors. Imaging findings demonstrating close proximity of the mass to the uterus, combined with a history of long-standing, slow growth, supported a benign uterine origin. Definitive diagnosis ultimately relied on postoperative histopathology. 3. Investigation of Venous Thromboembolism (VTE): The patient's acute onset of left lower extremity deep vein thrombosis (DVT) and pulmonary embolism (PE), in the context of prolonged bed rest (contributing to venous stasis, a component of Virchow's triad) and direct compression of the iliac vessels and inferior vena cava by the giant tumor (causing endothelial injury), was attributed to tumor-related secondary VTE. This reasoning effectively ruled out primary coagulation disorders or other precipitating factors.

**Figure 2:**
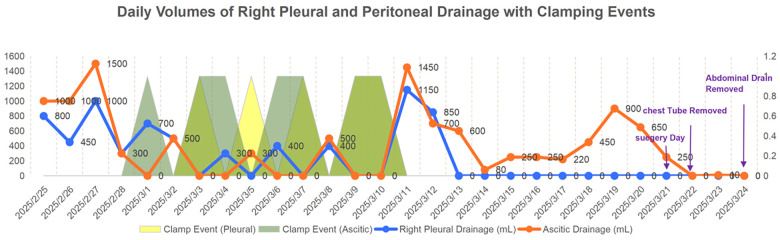
Summary of pleural and ascitic fluid drainage. The blue line represents the right pleural drainage volume, and the orange line represents the ascitic drainage volume. Light yellow translucent areas indicate pleural clamp events, and light green translucent areas indicate ascitic clamp events. Purple arrows mark special events (Surgery Day, Chest Tube Removal, Abdominal Drain Removal).

Through this systematic diagnostic approach, we ultimately integrated all complications into a core pathophysiological process—"biomechanical cascade triggered by a giant uterine leiomyoma"—and formulated a staged treatment strategy prioritizing hemodynamic stabilization followed by definitive surgical resection. The management was divided into two critical phases:

Phase 1: Preoperative resuscitation and preparation—The immediate priority was stabilizing vital signs. Ultrasound-guided thoracentesis and paracentesis were performed promptly to alleviate respiratory distress and abdominal compression. During the 24-day preoperative preparation period (25 days total), a cumulative total of 13,780 mL of pleural and ascitic fluid was drained, comprising 5,600 mL from the right pleural cavity and 8,180 mL from the peritoneal cavity. Postoperatively, pleural drainage ceased on day 2, and an additional 2,730 mL of ascitic fluid was drained before abdominal drain removal on day 4. The overall cumulative drainage throughout hospitalization reached 16,510 mL (Table 3, Figure 2). This aggressive preoperative decompression strategy effectively reduced intra-abdominal and intrathoracic pressure, optimized respiratory mechanics, and created a safe window for definitive surgery.

**Table 3:** Summary of clinical data.

Parameter	Preoperative	Intraoperative	Postoperative (Day 5)
Pleural Effusion Volume (ML)	5,600	0	Resolved
Ascites Volume (ML)	8,180	250	2,730
POP-Q Stage	IV	III	Stable, no progression
Tumor Size (cm)	27.5 × 19.1 × 23.1	26 × 14 × 24	Resected
Hernia Size (cm)	12 × 7 × 11	11 × 1.5 × 10	No recurrence

Given the presence of active deep vein thrombosis (DVT) and pulmonary embolism (PE) in a patient scheduled for major pelvic surgery, an inferior vena cava filter was placed on March 19, 2025, to prevent fatal intraoperative thromboembolism. Concurrently, aggressive parenteral nutritional support was administered to address severe hypoalbuminemia (nadir 18.3 g/L) and cachexia, elevating serum albumin to 33.7 g/L (Table 2) and optimizing the patient's condition for surgery.

On March 21, 2025, after thorough preoperative preparation, exploratory laparotomy was performed. Intraoperatively, a giant tumor measuring approximately 26 × 14 × 24 cm was identified, extending to the inferior border of the liver and adherent to the abdominal wall and portions of the bowel. A peritoneal defect of approximately 0.3 cm was noted at the umbilicus, corresponding to the preoperative umbilical hernia. Total hysterectomy with bilateral salpingo-oophorectomy and umbilical hernia repair were subsequently performed. The operative time was 2 h and 25 min, with an estimated blood loss of 600 mL and urine output of 200 mL; no blood transfusion was required. Intraoperative monitoring included invasive blood pressure (IBP), central venous pressure (CVP), BIS index, and serial blood gas analyses. Following uterine removal, the patient's blood pressure transiently dropped to 70/50 mmHg, which normalized after norepinephrine administration. Arterial blood gas analysis revealed pH 7.34 and BE −3, indicating mild metabolic acidosis, which resolved with appropriate management. Despite the presence of comorbidities, intraoperative hemodynamics remained generally stable.

Intraoperative frozen section suggested a smooth muscle tumor. Final paraffin section pathology confirmed a cellular leiomyoma with hemorrhage and edema, showing <5 mitotic figures per 10 high-power fields and no immunohistochemical features of malignancy.

**Figure 3:**
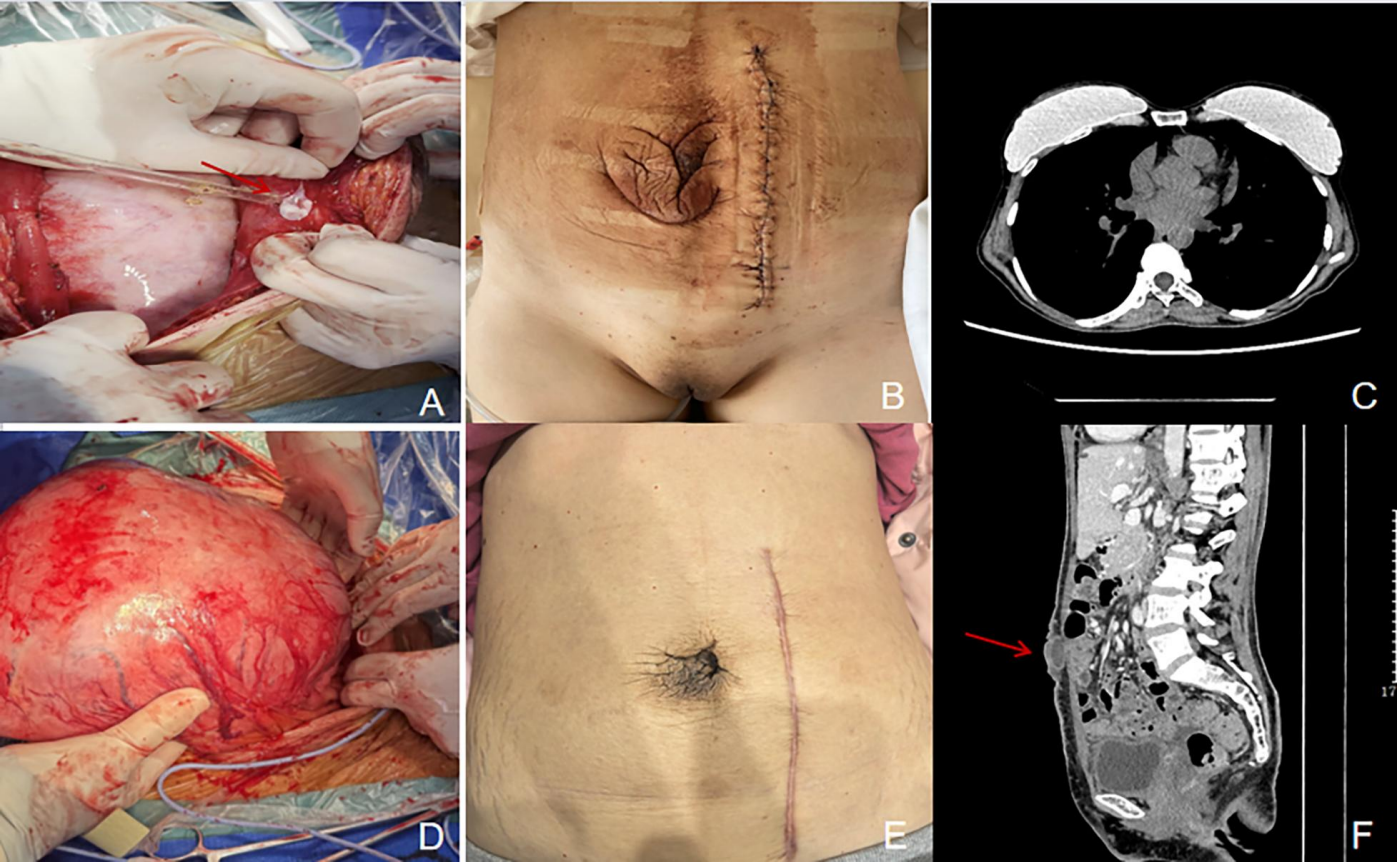
Postoperative follow-up imaging and intraoperative findings. **(A)** Intraoperative view showing the large fascial defect on the abdominal wall corresponding to the umbilical hernia (arrow). **(B)** Clinical photograph one week postoperatively, demonstrating significant retraction of the umbilicus and complete resolution of the posterior vaginal wall prolapse. **(C)** Axial chest CT image at one-month follow-up, revealing complete resolution of bilateral pleural effusions. **(D)** Intraoperative photograph of the giant uterine leiomyoma being delivered out of the abdominal cavity. **(E)** Clinical photograph at ten-month follow-up, showing near-normal restoration of umbilical anatomy without surgical repair. **(F)** Sagittal reconstruction of abdominal CT at three-month follow-up, showing a residual fascial defect at the umbilicus (red arrow), measuring approximately 36 × 11 mm.

Postoperative recovery was remarkable: pleural effusion and ascites completely resolved within 72 h, and CA-125 rapidly normalized. Anticoagulation therapy with rivaroxaban was continued postoperatively, resulting in a significant decrease in D-dimer levels. One-month follow-up CT demonstrated complete resolution of pleural effusion (Figure 3A). At three-month follow-up, vaginal prolapse showed no progression, umbilical hernia remained non-recurrent (Figure 3B), and lower extremity venous thrombosis was stable. The patient did not undergo additional pelvic floor reconstruction or umbilical plastic surgery. At nine-month follow-up, the umbilical morphology had substantially restored (Figure 3E).

## Discussion

This case is far from a simple giant leiomyoma; it demonstrates how a benign space-occupying lesion, when left uncontrolled, can trigger systemic catastrophe through purely physical and biomechanical mechanisms.

Pseudo-Meigs Syndrome (PMS) is a rare clinical syndrome characterized by “benign pelvic tumor + pleural and ascitic effusions + postoperative effusion resolution”, with an incidence <0.5%, and is often associated with benign ovarian tumors (1). This case of a 27.5 cm giant uterine leiomyoma as the primary cause of PMS, concurrently combined with umbilical hernia, POP-Q stage IV pelvic organ prolapse, and DVT/PE, is exceedingly rare in the literature (2). The long-term compression and elevated intra-abdominal pressure from the giant leiomyoma were the core triggers for the multi-system complications.

The significant CA125 elevation (370.0 U/mL) could initially be mistaken for ovarian malignancy. In PMS, CA125 elevation is linked to mechanical peritoneal irritation, mesothelial cell activation, and impaired lymphatic drainage (3). The trans-cavitary movement of fluid may occur via diaphragmatic defects or the abdominothoracic pressure gradient (4). Literature suggests combining cytological evaluation with a serum CA-125 to CEA ratio >25 to help diagnose the primary condition and exclude non-ovarian/fallopian tube/primary peritoneal cancers (5). In this case, the ratio was 462.5 (370.0/0.8), although significantly elevated, the serum-ascites albumin gradient (SAAG) of 0.87 g/dL and exudative characteristics (LDH 146 U/L) still supported a benign process, contrasting with the transudative nature of classic Meigs syndrome. CT initially suggested possible malignancy. Due to renal function limitations, contrast-enhanced MRI was not performed. The final diagnosis of benign cellular leiomyoma was based on postoperative pathology showing whorled architecture, good cellular differentiation, and <5 mitoses/10 HPF. It is important to note that giant leiomyomas carry a risk of smooth muscle tumors of uncertain malignant potential (STUMP), with 80% of STUMPs being >5 cm (6). Preoperative biopsy and extensive intraoperative frozen section sampling are crucial to avoid misdiagnosis.

Thrombosis Mechanism: In this case, compression of pelvic vessels by the giant leiomyoma (27.5 cm) was the core trigger for thrombosis. The tumor's sheer volume directly altered pelvic venous anatomy. CTV showed localized compression and narrowing of the inferior vena cava, while the left external iliac-femoral vein exhibited sluggish and turbulent flow due to persistent compression. Combined with immobility from prolonged bed rest, the mass effect caused vascular endothelial injury and a hypercoagulable state (elevated TAT, tPAIC) (7), fulfilling the key “stasis” mechanism of Virchow's triad. This compression not only caused left external iliac-femoral DVT but also led to PE due to thrombus dislodgement, forming a pathological chain of “compression-thrombosis-embolism”. Literature indicates that giant pelvic tumors are an independent risk factor for VTE, increasing the risk 3–5-fold compared to general gynecological patients. When tumor diameter exceeds 10 cm, the VTE incidence can be as high as 20% (7). This case, with a diameter of 27.5 cm, far exceeds the high-risk threshold, further underscoring the dominant role of compression.

Pelvic Floor Dysfunction: The co-existence of the giant leiomyoma, umbilical hernia, and vaginal prolapse highlights the close relationship between pelvic masses and pelvic floor dysfunction. The presentation resembled physiological changes seen in abdominal compartment syndrome: massive ascites and tumor growth increased abdominal volume, raising intra-abdominal pressure (IAP). Combined with 90% lung atelectasis and diaphragmatic elevation, IAP was likely >25–30 mmHg (8). The giant pelvic mass triggered a “biomechanical cascade”, ultimately causing multi-organ dysfunction. It disrupted pelvic support via a “triple-hit” mechanism: continuous high-pressure traction on the uterosacral-cardinal ligament complex leading to vaginal prolapse, distension of the umbilical ring forming a 12 × 7 × 11 cm hernial sac, and compression of the bladder neck causing urinary retention. Notably, prolapse symptoms improved significantly postoperatively without formal pelvic floor reconstruction, suggesting the potential for elastic recoil of pelvic floor structures after relief of the mass effect (9). This supports a delayed strategy of re-evaluating the need for pelvic floor reconstruction 3–6 months post-tumor resection, which may reduce risks of mesh infection and surgical complexity.

Multidisciplinary team (MDT) collaboration was pivotal to the successful management of this case. A team comprising 13 specialties delivered targeted interventions addressing the core challenges of thrombosis management, nutritional support, and surgical decision-making. Key interventions included transjugular inferior vena cava filter placement, intraoperative thromboelastographic monitoring, and early postoperative anticoagulation, which collectively resulted in a 76% reduction in D-dimer levels. Nutritional support employed a stratified approach, combining preoperative parenteral nutrition with albumin supplementation and gradual transition to enteral nutrition postoperatively. This strategy elevated serum albumin from a nadir of 26.6 g/L to 35.2 g/L by postoperative day 5. Based on a POSSUM score of 49 (indicating high surgical risk), we adopted a staged surgical strategy—consisting of hysterectomy with umbilical hernia repair in the first phase and elective pelvic floor reconstruction in the second—to balance therapeutic efficacy with patient safety.

This case underscores the critical importance of early detection and timely intervention for uterine leiomyomas. While small, asymptomatic fibroids may be managed conservatively with surveillance, surgical intervention should be actively considered in the following scenarios:

Rapid growth or significant enlargement: Fibroids exceeding 5 cm in diameter or demonstrating rapid growth on serial imaging studies carry an increased risk of malignant transformation and complications. This risk was exemplified in the present case, where the leiomyoma rapidly progressed from 8 cm to 27.5 cm.

Symptomatic presentation: Surgical intervention is indicated for fibroids causing menorrhagia with consequent anemia, chronic pelvic pain, severe abdominal distension, or compressive symptoms such as urinary frequency, defecatory difficulty, or pelvic organ prolapse. The presence of severe pelvic organ prolapses and compressive symptoms in this case represented clear surgical indications.

Giant fibroids: Leiomyomas exceeding 10 cm in diameter are associated with significantly elevated risks of complications, including degeneration, torsion, sarcomatous transformation, and biomechanical decompensation—as demonstrated by the multisystem failure observed in this case.

Suspected malignancy: Surgical resection is warranted when imaging suggests possible malignancy or when considering smooth muscle tumors of uncertain malignant potential (STUMP).

This case serves as a reminder that even benign leiomyomas, if allowed to progress unchecked, can culminate in life-threatening complications. Proactive management, thorough patient education, and timely referral to surgical specialists are paramount.

## Patient perspective

The patient was interviewed postoperatively to obtain her perspective on the disease trajectory and treatment experience. She expressed profound regret for delaying surgical intervention despite repeated medical recommendations over the four-year period. She described feeling gradually overwhelmed by her symptoms—progressive abdominal distension, dyspnea, and immobility—yet her personal beliefs and distrust of medical intervention prevented her from seeking timely care.

Upon admission, she recalled feeling frightened and confused by the severity of her condition, which contributed to her initial resistance to treatment. However, as her symptoms rapidly improved following drainage and nutritional support, she gradually developed trust in the medical team. She described the preoperative preparation period as “physically challenging but necessary” and expressed gratitude for the multidisciplinary team that managed her complex condition.

Postoperatively, she reported a dramatic improvement in her quality of life. At discharge, she was able to breathe comfortably, walk independently, and perform daily activities—functions she had lost for months prior to admission. She has now returned to her pre-illness state and is able to live a normal life. She remains under regular follow-up and, given the significant symptomatic improvement, has declined additional reconstructive surgery for pelvic floor prolapse and umbilical plastic surgery. She hopes that sharing her experience will encourage other patients with uterine leiomyomas to seek timely medical advice and adhere to treatment recommendations.

## Conclusion

In summary, this case report describes an extremely complex biomechanical imbalance of the pelvis and abdomen triggered by a giant cellular uterine leiomyoma, which rarely manifested concurrent pseudo-Meigs syndrome and a triad of “umbilical hernia-pelvic organ prolapse-venous thromboembolism” induced by sustained intra-abdominal hypertension. The patient faced multiple threats including respiratory failure, urinary retention, severe malnutrition, and life-threatening pulmonary embolism. This case serves as a warning to clinicians to attach great importance to early intervention for giant pelvic tumors and to fully recognize their potential to trigger systemic chain reactions through mechanical compression. Simultaneously, it demonstrates that through structured MDT collaboration and individualized staged treatment, a successful outcome is achievable even in such critical and complex cases. For healthcare professionals worldwide, this report reinforces the indispensable value of integrated medicine in managing complex cases.

The management of uterine leiomyomas requires a nuanced strategy that balances expectant observation for small, asymptomatic lesions with timely surgical intervention for symptomatic, rapidly growing, giant, or suspected malignant tumors. When indicated, early surgical intervention is crucial for preventing the development of severe complications—including pseudo-Meigs syndrome, pelvic organ prolapse, herniation, and venous thromboembolism—thereby safeguarding patient health and averting life-threatening sequelae.

## Data Availability

The raw data supporting the conclusions of this article will be made available by the authors, without undue reservation.
